# S05-4 Progressive muscle-strengthening exercise is feasible among older people in the ‘Strength in Old Age Program'

**DOI:** 10.1093/eurpub/ckac093.026

**Published:** 2022-08-29

**Authors:** Heli Starck, Pirjo Kalmari, Eerika Saloranta, Katja Borodulin

**Affiliations:** Age Institute, Helsinki, Finland

**Keywords:** physical activity, older persons, implementation, volunteers, participation

## Abstract

Strong legs and good balance increase mobility and prevent falls in old age. Gym exercise is an effective way to improve muscle strength in old age, particularly when organized in a progressive training program. Current challenge is to recognize and motivate older people to join muscle-strengthening exercise groups. The national “Strength in Old Age Program” (2005-) aims to launch guided strength and balance exercises for independently living older adults with decreased functional capacity. One core activity is to find and motivate old people to participate in a progressive 8-12-week muscle strengthening exercise program. We aimed to increase participation in the organized activity and support participation after the progressive program had ended, to improve the exercise facilities at local level, and to study the perceived benefits from the participants in the Program. Setting was real-life setting, i.e. no case-control trials were set. Good practices were developed to recognize and recruit participants for the program: perform functional capacity tests in locations where old people go as part of their routines, such as free flu vaccination events, use screening questionnaires systematically for all, and activate/guide health care professionals to refer the high-risk patients to gym training. Further, as many municipalities were limited in their gym facilities, the program developed the following solutions: collaborate with private sector service providers, purchase only the essential leg-strengthening devices and use cheap solutions, improve the quality of training by increasing training frequency and combine nutritional advice. The program aimed to improve sustainability of training, where several approaches were found successful: educate peers who instruct gym groups in future, develop the chain of professional exercise counselling from health care to sport sector, and provide free-of-charge gym training sessions to continue after their progressive program. The participants (Survey of exercise group participants, n = 1022) of the progressive gym training program perceived mostly good or strong benefits from their participation in the program. Participants who attended gym training felt the highest impact on muscle strength, fitness and activities of daily living, when compared to other types of training groups (eg balance training) in the program.

Conclusion suggests that gym exercise is a feasible good practice that should be encouraged in real-life setting.

